# Biomimetic Hydrodynamic Sensor with Whisker Array Architecture and Multidirectional Perception Ability

**DOI:** 10.1002/advs.202405276

**Published:** 2024-08-09

**Authors:** Huangzhe Dai, Chengqian Zhang, Hao Hu, Zhezai Hu, Haonan Sun, Kan Liu, Tiefeng Li, Jianzhong Fu, Peng Zhao, Huayong Yang

**Affiliations:** ^1^ The State Key Laboratory of Fluid Power and Mechatronic Systems Zhejiang University Hangzhou 310027 China; ^2^ The Key Laboratory of 3D Printing Process and Equipment of Zhejiang Province College of Mechanical Engineering Zhejiang University Hangzhou 310027 China; ^3^ Center for X‐Mechanics Department of Engineering Mechanics Zhejiang University Hangzhou 310027 China

**Keywords:** Biomimetic sensors, hydrodynamic perception, magnetic soft sensors, underwater electronics, whisker array

## Abstract

The rapid development of ocean exploration and underwater robot technology has put forward new requirements for underwater sensing methods, which can be used for hydrodynamic characteristics perception, underwater target tracking, and even underwater cluster communication. Here, inspired by the specialized undulated surface structure of the seal whisker and its ability to suppress vortex‐induced vibration, a multidirectional hydrodynamic sensor based on biomimetic whisker array structure and magnetic 3D self‐decoupling theory is introduced. The magnetic‐based sensing method enables wireless connectivity between the magnetic functional structures and electronics, simplifying device design and endowing complete watertightness. The 3D self‐decoupling capability enables the sensor, like a seal or other organisms, to perceive arbitrary whisker motions caused by the action of water flow without complex calibration and additional sensing units. The whisker sensor is capable of detecting a variety of hydrodynamic information, including the velocity (RMSE < 0.061 m s^−1^) and direction of the steady flow field, the frequency (error < 0.05 Hz) of the dynamic vortex wake, and the orientation (error < 7°) of the vortex wake source, demonstrating its extensive potential for underwater environmental perception and communication, especially in deep sea conditions.

## Graphical abstract

Mimicking the specialized undulated surface structure of the seal whisker, the sensor is designed based on 3D self‐decoupling displacement sensing of the centripetally magnetized magnetic film. It can measure the velocity and direction of steady‐state flow fields as well as perceive the frequency and orientation of dynamic vortex wakes accurately, avoiding possible watertight problems through its magnetic wireless penetration characteristics.

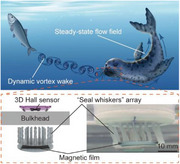

## 1 Introduction

With increasing attention to ocean resources, in recent years, a variety of underwater robots, such as autonomous underwater vehicles (AUVs)^[^ ^1^, ^2^ ^]^ and underwater bionic robots,^[^ ^3^, ^4^ ^]^ have been rapidly developed to explore and monitor marine information, whose development trend of intelligence,^[^ ^5^ ^]^ miniaturization, clustering,^[^ ^6^ ^]^ and deep sea^[^ ^7^ ^]^ put forward higher requirements for the perception capability of the underwater environment information. For example, the perception of hydrodynamic information such as flow velocity and vortex wake provides the key technical basis for navigation, tracking, and even underwater communication of underwater equipment (in particular, small ones that cannot carry large locating and sensing devices). Underwater visual perception^[^ ^8^, ^9^ ^]^ based on optics and underwater auditory perception^[^ ^10^, ^11^ ^]^ based on ultrasound are commonly used in practical applications, among which, especially, sonar technology has been quite mature. However, they are difficult to apply to these rapid‐developed small clustered equipments due to their substantial size, weight, redundant add‐ons, prohibitive cost, and great power consumption. Therefore, there is an urgent need for a hydrodynamic sensor with a simple structure. As a novel underwater environment perception technology, underwater tactile perception^[^ ^12^, ^13^ ^]^ has been widely concerned by researchers, most of whom have focused on bionic tactile perception technology. Cilia play an important role as tactile sensing organs in many organisms,^[^ ^14^ ^]^ including the human body.^[^ ^15^ ^]^ Mimicking the basic structure of cilia, many studies have achieved important perceptual tasks, such as tactile perception^[^ ^16^, ^17^ ^]^ and flow detection,^[^ ^18^, ^19^ ^]^ some of which have even equipped the “cilia” with driving schemes to achieve actuation‐enhanced multifunctional sensing.^[^ ^20^ ^]^ The lateral line is a fish‐specific ciliated organ that assists the fish in preying or avoiding potential danger. A large number of underwater robots use artificial lateral lines as underwater tactile sensing organs to perceive their own motion state,^[^ ^21^, ^22^ ^]^ obtain external interference,^[^ ^23^ ^]^ and locate vibration sources.^[^ ^24^, ^25^ ^]^ However, the cylindrical cilia structure will be affected by the Karman vortex street effect^[^ ^26^ ^]^ in the flow field, which will lead to vortex‐induced vibrations (VIVs) and decrease the stability and accuracy of the sensor signal.

**Figure 1:**
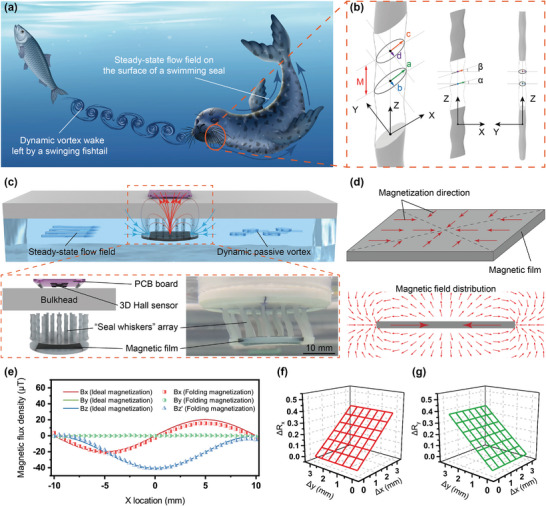
Design of the whisker sensor for multidirectional hydrodynamic perception. a) Illustration of the seal sensing its own movements and tracking fish underwater. b) 3D structure diagram of the seal whisker. c) Top: Rendering diagram of the whisker sensor sensing steady‐state flow fields and dynamic vortex wakes. Bottom left: Exploded view of the whisker sensor. Bottom right: Experimental photo of the whisker sensor under steady‐state flow field. d) Diagram of the film's folding magnetization and profile of the magnetic field distribution of the centripetally magnetized film. e) The 3D magnetic field distribution comparison below the film along the *X*‐axis between the ideal magnetization and the folding magnetization. f) The relationship between the decoupling parameter *R_x_* and the displacements of the magnetic film in the *x* and *y* directions. g) The relationship between the decoupling parameter *R_y_* and the displacements of the magnetic film in the *x* and *y* directions.

By virtue of their own cilia (whiskers), the seal can not only sense their own motion state underwater but also catch the vortex wake generated by prey to track it,^[^ ^27^, ^28^, ^29^ ^]^ as shown in **Figure** **1**a. Figure 1b shows the unique shape of the seal whisker.^[^ ^30^ ^]^ This specialized undulated surface structure enables the whisker to suppress VIVs, that is, to eliminate most of the self‐induced noise, thus improving its sensitivity to the vortex wake generated by prey. The impressive ability has brought the seal whisker to the attention of the scientific community.^[^ ^31^ ^]^ Just as whiskers can't sense without the tactile cells around their roots, force sensors are also necessary as the bases of artificial whiskers. Consequently, many artificial seal whiskers have been studied in combination with tactile sensors of various principles, such as piezoelectricity,^[^ ^32^ ^]^ piezoresistive,^[^ ^33^, ^34^ ^]^ triboelectric,^[^ ^35^ ^]^ and so on. However, because of the single perception dimension of these tactile sensors, cilia (whisker) sensors can only perceive either the steady‐state flow field or the dynamic vortex wake directionally, rather than both and multi‐directionally, like seal whiskers. It's worth mentioning that magnetic‐based tactile sensors have many advantages, such as simple structure, high physical robustness, low cost, and wireless penetrability,^[^ ^36^, ^37^, ^38^ ^]^ thus being applied in numerous industries.^[^ ^39^, ^40^ ^]^ Recently, designable magnetization^[^ ^41^, ^42^ ^]^ enabled by flexible magnetic materials^[^ ^43^, ^44^ ^]^ and newly developed algorithms^[^ ^45^ ^]^ have contributed to multi‐dimensional force perception^[^ ^46^, ^47^ ^]^ and even multi‐dimensional self‐decoupling perception (Force measurements in 2D^[^ ^48^ ^]^ or 3D^[^ ^49^ ^]^ directions are independent of each other.) of magnetic tactile sensors, bringing them back into the researchers' sight. Therefore, the combination of artificial seal whiskers and the 3D decoupling magnetic tactile sensors would be a creditable attempt, which allows the whisker sensor to perceive the whisker's movements in arbitrary directions, like a seal, obtaining multidirectional hydrodynamic information including steady flow field and dynamic vortex wake. Moreover, the wireless penetrability of magnetic‐based tactile sensing can be fully utilized in underwater applications, completely avoiding the watertightness problems caused by leads and perforations, which is extremely critical for sensing applications in extreme deep oceans. Meanwhile, the inherent watertightness and resistance to water pressure also eliminate the additional volume and weight added by the pressure‐resistant packaging of the related devices.

In this article, we report a flow sensor with 3D magnetic‐based force‐decoupling perception capability and a bionic seal whisker array structure that can realize the velocity and direction measurement of a multidirectional steady‐state flow field as well as the frequency and direction measurement of a multidirectional dynamic vortex wake. Consistent with the basic structure of a magnetic tactile sensor, our whisker sensor is composed of a magnet (a centripetally magnetized film), an elastic deformable layer (a seal whisker‐inspired silicone cilia array), and a magnetic sensing unit (a 3D Hall sensor). Through a 3D self‐decoupling theoretical model, the spatial triaxial displacement of the centripetally magnetized film could be calculated according to the triaxial magnetic flux density data of the Hall sensor, reflecting the hydrodynamic information received by the whisker array. In addition to laying the foundation for the 3D self‐decoupling theory, the centripetal magnetization arrangement also strengthened the magnetic field and enabled an elongation of the working distance between the magnetic film and the Hall sensor, thereby ensuring the separate installation of electrical parts that need to be insulated from water and deformed parts under water. Mimicking the specialized undulated surface structure of the seal whisker, the artificial whisker array still equipped the whisker array with a considerable ability to suppress vortex‐induced vibration even while ensuring the uniformity of multidirectional measurement through the centripetal arrangement. It nearly doubled the stability of flow rate measurement compared with the cylindrical cilia array. The whisker sensor was rotated in an annular flow channel to simulate the perception of multidirectional steady flow fields and demonstrate the strong capability of sensing their velocity. Furthermore, the frequency of the underwater vortex wake generated by a swinging silicone fishtail and its orientation could be accurately sensed by our sensor. The artificial whisker sensor based on magnetic 3D self‐decoupling is a significant approach for multidirectional hydrodynamic perception, whose powerful sensing capability and wireless penetration characteristic will greatly elevate its application potential in underwater sensing and communication.

## 2 Results and Discussion

### 2.1 Mechanism and Model of the Multidirectional Perception

The whisker sensor for sensing steady‐state flow fields and dynamic vortex wakes is of a sandwich‐like and multi‐layered structure, as shown in the top and bottom left of Figure 1c. The bottom layer (thickness 1.4 mm) is the magnetic film (20 mm × 20 mm × 1 mm) encased in silicone elastomer, which is the component that generates the magnetic field. The middle layer is an elastic whisker array (thickness 10.92 mm), which is the main component of deformation caused by the water flow. The top layer, installed on the other side of the bulkhead and separated from the above components, is a 3D Hall sensor (MLX90393, Melexis, Belgium) that measures the spatial magnetic field generated by the magnetic film. As shown in the bottom right of Figure 1c, when the whisker array is deformed by the impact and viscosity of the water flow, the position of the magnetic film will be shifted with the array. And the spatial magnetic field generated by the magnetic film will change accordingly. The 3D Hall sensor will detect these magnetic flux density changes, which could be analyzed using the 3D self‐decoupling model to obtain the triaxial displacement information of the magnetic film.

The 3D self‐decoupled model is based on an ideal magnetic film, whose thickness is *d*, lying in the *x–y* plane, and whose magnetization arrangement is the superposition of two orthometric sinusoids in the *x–y* plane:

(1) $$m_{x}=m_{0}\sin\left(kx\right),\cdot m_{y}=m_{0}\sin\left(ky\right),\cdot m_{z}=0$$

where *k* = 2*π*/*λ* is the wavenumber, *m_0_* is the maximum magnitude of each component. According to the magnetic properties of the 2D sinusoidal magnetization arrangement,^[^ ^50^ ^]^ the 3D magnetic flux density at different positions generated by the magnetic film can be calculated as follows:

(2) $$\left\{\begin{array}{c} \;B_{x}\;=\;\;-Ke^{kz}\sin\left(kx\right)\\ \;B_{y}\;=\;\;-Ke^{kz}\sin\left(ky\right)\\ \;B_{z}\;=\;\;Ke^{kz}\left(\cos\left(kx\right)\;+\;\cos\left(ky\right)\right) \end{array}\right.$$

where $K\;\;=\;\frac{1\;-\;e^{-kd}}{2}\;m_{0}$. Through trigonometric and substitution operations, we could obtain the relationship between the 3D coordinate position (*x*, *y*, *z*) below the magnetic film and its triaxial magnetic flux density (*B_x_*, *B_y_*, *B_z_*):

(3) $$\left\{\begin{array}{c} \;\tan\left(kx\right)\;=\;\;-\frac{B_{x}}{B_{z}\;-\;\frac{B_{z}^{2}\;-\;\left(B_{y}^{2}\;-\;B_{x}^{2}\right)}{2B_{z}}}\\ \;\tan\left(ky\right)\;=\;-\frac{B_{y}}{B_{z}\;-\;\frac{B_{z\;}^{2}+\;\left(B_{y}^{2}\;-\;B_{x}^{2}\right)}{2B_{z}}}\\ K\;e^{kz}\;=\;\sqrt{{\left(Bz\;-\;\frac{B_{z}^{2}\;+\;\left(B_{y}^{2}\;-\;B_{x}^{2}\right)}{2B_{z}}\right)}^{2}\;+\;B_{y}^{2}}\; \end{array}\right.$$

Thus, the displacement of the magnetic film could be calculated according to the magnetic flux density signals obtained by the Hall sensor. Here, we introduced three decoupling parameters (*R_x_*, *R_y_*, and *S_z_*), which are linearly related to the triaxial displacement, respectively, reflecting the magnetic film displacement more intuitively to obtain hydrodynamic information more accurately:

(4) $$\left\{\begin{array}{c} \;R_{x}\;=\;\arctan\left(\frac{B_{x}}{B_{z}\;-\;\frac{B_{z}^{2}\;-\;\left(B_{y}^{2}\;-\;B_{x}^{2}\right)}{2B_{z}}}\right)\;\\ \;R_{y}\;=\;\arctan\left(\frac{B_{y}}{B_{z}-\frac{B_{z}^{2}\;+\;\left(B_{y}^{2}\;-\;B_{x}^{2}\right)}{2B_{z}}}\right)\;\\ \;S_{z}\;=\;\ln\left(\sqrt{{\left(Bz\;-\;\frac{B_{z}^{2}\;+\;\left(B_{y}^{2}\;-\;B_{x}^{2}\right)}{2B_{z}}\right)}^{2}\;+\;B_{y}^{2}}\right)\; \end{array}\right.$$

where *B_x_*, *B_y_*, and *B_z_* are the magnetic flux densities along the *x*, *y*, and *z* directions, respectively. According to the 3D self‐decoupling model (Equations (3) and (4)), the decoupling parameter *R_x_* is independently related to the magnetic film's displacement in the *x* direction, while the decoupling parameters *R_y_* and *S_z_* are independently related to the magnetic film's displacements in the *y* and *z* directions, respectively, that is, when the magnetic film only generates displacement in the *x* direction, only the decoupling parameter *R_x_* will change, while the decoupling parameters *R_y_* and *S_z_* remain constant; when the magnetic film only generates displacement in the *y* direction, only the decoupling parameter *R_y_* will change, while the decoupling parameters *R_s_* and *S_z_* remain constant; when the magnetic film only generates displacement in the *z* direction, only the decoupling parameter *S_z_* will change, while the decoupling parameters *R_x_* and *R_y_* remain constant. “3D self‐decoupling” means that the perception and calculation of the triaxial displacement of the magnetic film do not affect each other, which is the theoretical basis for using one sensing unit to realize multidirectional hydrodynamic perception.

The above 3D self‐decoupling model was derived from an ideal magnetic film with infinite period, area, and centripetal sinusoidal magnetization mode, none of which could be achieved in practical experiments. Therefore, we designed a single‐period centripetally magnetized film with a similar folding magnetization arrangement to replace this ideal magnetic film, as shown in Figure 1d, where the magnetic field generated by the magnetic film with this magnetization arrangement is also shown. We have proved that this replacement is reasonable and effective in this working range.^[^ ^49^ ^]^ As long as the corresponding linear correction of the magnetic flux density in the *z*‐direction (*B_z_*) is carried out, the spatial distribution of the triaxial magnetic flux density of the two kinds of magnetic films will tend to be consistent within a certain range (Figure 1e), and thus the 3D self‐decoupling model is still valid. The linear corrections for *B_z_* in the decoupling parameters *R* (*R_x_* and *R_y_*) and *S* (*S_z_*) are *B_z_'*  = *k_1_B_z_ *+ *c_1_* and *B_z_“” *= *k_2_B_z_ *+ *c_2_*, respectively (*k_1_*, *c_1_*, and *k_2_*, *c_2_* are the correction coefficients of *B_z_* in *Rx*, *Ry*, and *Sz*, respectively. *k_1_ = 1.63; c_1_  = 164.38; k_2 _= 0.89; c_2_  = −0.27*).

Under the action of the steady‐state flow field, the whisker array will drive the magnetic film to produce shear displacement along the direction of water flow. While under the action of the dynamic vortex wake, the whisker array and the magnetic film will produce shear oscillation perpendicular to the direction of the vortex wake together. Through this 3D self‐decoupling model, the shear decoupling parameters *R_x_* and *R_y_* can be calculated by the triaxial magnetic flux density signals obtained by the Hall sensor. As shown in Figure 1f,g, the calculated decoupling parameters *R_x_* and *R_y_* are independently related to the displacements of the magnetic film in the x and y directions, respectively. Consequently, the shear displacement of the magnetic film can be obtained in real time according to the shear decoupling parameters, thereby realizing the hydrodynamic perception of the multidirectional steady‐state flow fields and the dynamic vortex wakes.

### 2.2 Design and Optimization of the Whisker Array Architecture

**Figure 2:**
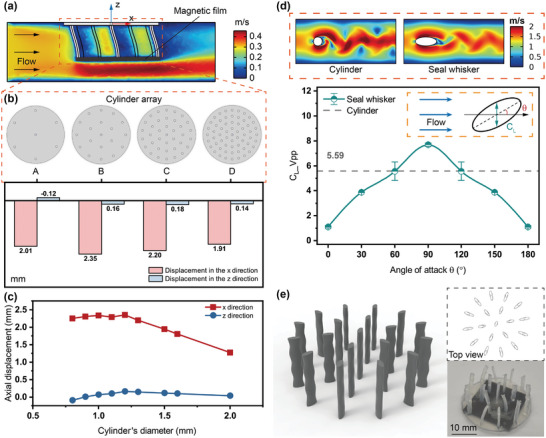
Structure design and optimization of the whisker array. a) Fluid‐structure coupling simulation (COMSOL Multiphysics) of the cylinder array sensor. b) Four kinds of cylinder arrays and the simulated axial displacement of the magnetic films below them. c) The relationship between the diameter of the cylinder in the array of type B and the simulated axial displacement of the magnetic film. d) The suppression effect of the seal whisker on VIVs and the influence of the whisker's angle of attack. e) The finalized centripetal seal whisker array and the photo after embedding the magnetic film.

The key component of the whisker sensor for hydrodynamic perception is the whisker array, which determines the sensitivity and stability of the perception. The overall size of the whisker array is determined by the size of the magnetic film, while the number and diameter of the whiskers still greatly affect the perceptual sensitivity of the sensor, which needs to be further optimized. As shown in **Figure** **2**a, without considering the influence of the whisker shape, the fluid‐structure coupling simulation of the whisker sensor was carried out in COMSOL Multiphysics, using the cylinder as the shape basis of the array optimization. In the simulation, a 0.3 m s^−1^ water flow in a channel (70 mm × 50  ×  mm  ×  20 mm) was used as the steady‐state flow field. The array sensor was fixed in the center at the top of the channel. The displacement of the magnetic film was recorded as a measure of the sensor sensitivity. Four kinds of arrays with different numbers of cylinders (A, B, C, and D) are shown in Figure 2b, all of which are evenly arranged in circles to maximize the uniformity of multidirectional measurements. Each cylinder is simultaneously subjected to the combined action of the water flow force, the deformation resistance, and the pull generated by the gravity of the magnetic film. When the number of cylinders increases, the resistance of the magnetic film's gravity decreases after equalization, while the effect of blocking each other between the cylinders is enhanced, and the water flow force is decreased too. The histogram shows the magnetic film displacements of four arrays under the same flow field. Compared to the displacement in the *x* direction, the magnetic film's displacement advantage of array B in the *z* direction is more obvious, which will be amplified in practical applications due to the actual complex effects of turbulence. When the number of cylinders and their respective positions are constant, the water flow force on the cylinder will increase with the increase in its diameter, but at the same time, the deformation of the cylinder itself becomes more difficult. The relationship between the diameter of the cylinder in the array of type B and the simulated axial displacement of the magnetic film is shown in Figure 2c, which indicates that array B has the greatest sensitivity when the cylinder diameter is ≈1.2 mm.

As shown in the planar fluid simulation diagram in the upper part of Figure 2d, alternating vortices (Karman vortex street) will be generated downstream as the water flow passes through the cylinder. When the vortices break away from the surface of the cylinder, they will exert unstable forces on the surface, resulting in VIVs, which lead to fluctuations in the magnetic flux density signals and the inaccurateness of the flow velocity measurement for our whisker sensor. Compared with the circular section, the vortex street generated by the elliptic section under the same flow field is more stable and leads to smaller VIVs, which indicates that the seal whisker's specialized undulated surface structure has a good suppression effect on VIVs. Here, we used the lift coefficient *C_L_* to evaluate the oscillation force perpendicular to the flow direction, taking the major axis, *2a*, of the elliptic cylinder as the representative length:^[^ ^51^, ^52^ ^]^

(5) $$C_{L}\;=\;\frac{F_{L}}{\frac{1}{2}\rho U^{2}\left(2a\right)}\;$$

where *F_L_* is the lift force, *U* is the flow velocity, and *2a* is the length of the major axis of the ellipse. This lift coefficient is related to the Reynolds number and the shape of the object, but not the size of the object, so we enlarged the size of the ellipse section (≈8 times, *a* = 4.8 mm, *b *= 2 mm) to facilitate simulation. In addition, because the vortices generated in the simulation are not completely symmetrical, we calculated the average of the peak‐to‐peak values of the lift coefficient (*C_L__Vpp*) after the stabilization of the vortex street for comparison. As shown in the curve graph in Figure 2d, with the increase of the ellipse's angle of attack in the flow field, the peak‐to‐peak value of the lift coefficient also increases, indicating that the vortex‐induced force of the ellipse cylinder also increases. The gray dashed line shows the peak‐to‐peak value level of the lift coefficient of the cylinder (radius: 2 mm) at the same flow field, which shows that the VIVs of the seal whisker with an elliptical section are smaller than that of the cylinder at most angles of attack. Therefore, we could arrange the thin rods with the seal whisker structure in a way that the major axes are aligned with the center. With this design, the whisker sensor not only has a good overall VIV suppression ability but also greatly improves the uniformity of omnidirectional perceptual sensitivity.

To sum up, we took 1.2 mm as the maximum minor axis size of the seal whisker (size *2d* = 1.2 mm in Figure 1b), that is, doubled the original size of the idealized seal whisker model^[^ ^30^ ^]^ (Table S1, Supporting Information). Three feature shapes were taken as the complete whisker (length: *6* *M = *10.92 mm). The major axes of all whiskers were aligned with the center for the centripetal arrangement in the method of array B. (The radii of the first and second whisker circles are 6.7 and 13.4 mm, respectively and the whiskers are evenly distributed in the circle at equal angles.) The finalized centripetal seal whisker array and the photo after embedding the magnetic film are shown in Figure 2e. Please refer to Table S2 (Supporting Information) to obtain other related parameters of the finalized whisker sensor.

### 2.3 Perception of Multidirectional Steady‐State Flow Field

**Figure 3:**
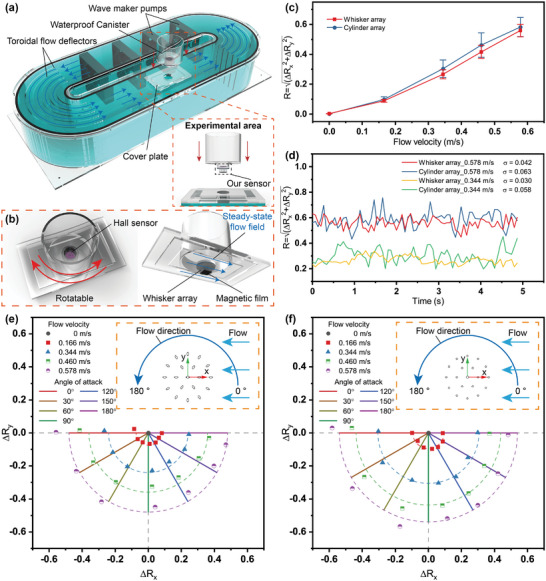
Perception of multidirectional steady‐state flow fields by our sensor. a) Device of the annular steady‐state flow field generator. (The dashed box shows the experimental area where our sensor is installed.) b) Schematic diagram of the experimental device for sensing multidirectional steady‐state flow fields by the sensor. c) The relationship between the shear decoupling parameter *R* and the flow velocity. (The flow direction is 0^○^.) d) Comparison of flow velocity sensing signal stability between cylinder array and whisker array. e) Perception of multidirectional steady‐state flow fields by the centripetal seal whisker sensor. f) Perception of multidirectional steady‐state flow fields by the cylinder array sensor.

One of the most important elements of hydrodynamic information is the velocity of the flow field. Therefore, the perception of flow velocity is one of the basic functions of hydrodynamic sensors. As shown in **Figure** **3**a (Figure S1, Supporting Information), we built a closed annular water tank using acrylic plates, which was capable of generating a stable annular flow field driven by four wave maker pumps (MOW‐22, Jebao Corp., Guangdong, China). The pumps and the experimental area were located in two opposite straight channels, and the toroidal flow deflectors were also installed in the bend, so as to minimize the disturbance of the vortexes generated by the blades of the wave maker pumps on the steady‐state flow field. The pumps' power could be controlled to adjust the flow field velocity (≈0 to 0.6 m s^−1^). The dashed box shows the experimental area for multidirectional steady‐state flow field perception. Our sensor was installed at the bottom of a cylindrical waterproof canister, which could be immersed into the water in the annular flow channel through the hole in the cover plate for the velocity sensing experiment, as shown in Figure 3b. The Hall sensor and the whisker array were mounted on either side of the canister bottom (thickness: 3 mm; the distance between the magnetic film and the Hall sensor is 18.92 mm.). Moreover, the canister was rotatable so that the whisker sensor could be subjected to the actions of the flow fields in different directions in the same unidirectional flow field generator.

As mentioned above, the magnitude of the shear decoupling parameters reflects the magnitude of the shear displacements of the magnetic film, which is closely related to the flow field velocity. Therefore, *R* (*R* ^2^  =  *R_x_* ^2^  +  *R_y_* ^2^) was introduced as a direct measure of the flow velocity. In addition, we used the cylinder array sensor as a control group to carry out steady‐state flow field sensing experiments to demonstrate the structural advantages of the seal whisker array. Figure 3c shows the relationship between parameter *R* and flow velocity under the action of a steady‐state flow field in the direction of 0^○^. The actual velocity of the flow field was measured by the current meter (LS300‐A) at the same position in the flow channel. It can be seen that the velocity sensing sensitivity of the whisker sensor and the cylinder array sensor differs not significantly, while the error bar of signal *R* when using the cylinder array is almost twice that when using the whisker array, which indicates that the whisker sensor has higher signal stability. The output signals (parameter *R*) of the two sensors during steady‐state flow field perception were collected to compare the stability of the signals at two flow velocity levels (Figure 3d). Here, we used the standard deviation (σ) to evaluate the stability of the signal. Compared with the cylinder array, the whisker sensor has smaller signal fluctuations under the action of both high and low flow velocities. Therefore, it has a higher perceived stability of a steady‐state flow field, especially at low velocity levels. In addition, the RMSE (root‐mean‐square error) of the whisker sensor in measuring the velocity of the unidirectional steady‐state flow field is less than 0.061 m s^−1^.

One of the important advantages of our whisker sensor is the ability to perceive the direction and magnitude of the flow field through only one 3D Hall sensor. By rotating the waterproof canister, the sensor could be rotated together in a directional flow field, thus changing the flow direction of the flow field relative to the sensor for experimental testing. Five velocity levels were used in each flow direction to verify the feasibility of multidirectional flow velocity sensing. Figure 3e shows the results of sensing the flow field by our sensor at different angles of attack, where the horizontal and vertical coordinates represent the variations of decoupling parameters *R_x_* and *R_y_* relative to the initial position, respectively, directly reflecting the displacement changes of the magnetic film along the *x* and *y* directions. The sensing points of the same flow velocity at different angles of attack almost show a semicircular distribution, and the spacing of each velocity level is basically consistent, which indicates that it has strong capability and high uniformity for multidirectional flow velocity measurement at each angle of attack. In addition, the sensing points in the same flow direction provide excellent straight line fit and are all close to the standard direction, which demonstrates the sensor's strong repeatability and acceptable accuracy of flow direction perception. However, it can't be ignored that there are still certain errors in the measured angles of attack, which may be attributed to the deflection angle between the direction of the rotatable waterproof canister and the actual direction of the flow field. In addition, manufacturing errors such as glue bonding thickness, whisker angle distortion, etc. may also lead to the deviation of the measurement angles. The same experiments were carried out for the cylinder array sensor, as shown in Figure 3f. Compared with the whisker sensor, the distribution of the cylinder array sensor's velocity‐sensing points is more chaotic and irregular, and the measurement angle deviation is larger. As with the whisker array, the reasons for these problems also include manufacturing and assembly errors, but the larger fluctuations of the perceived signals may more easily contribute to the reductions in the uniformity of the velocity measurement and the repeatability of the angle measurement.

It can be concluded that the whole whisker array still had a remarkable effect on suppressing VIVs in the actual steady‐state flow field sensing experiment after the elliptic whiskers were arranged centripetally. The signal fluctuation was reduced by about half compared with the cylinder array. Moreover, the centripetal arrangement endowed the whole whisker array with a good unity for the velocity measurement of multidirectional flow fields, equal to or even better than the cylinder array, which is conducive to the rapid calibration and practical application of the sensor.

### 2.4 Perception of Multidirectional Dynamic Vortex Wake

**Figure 4:**
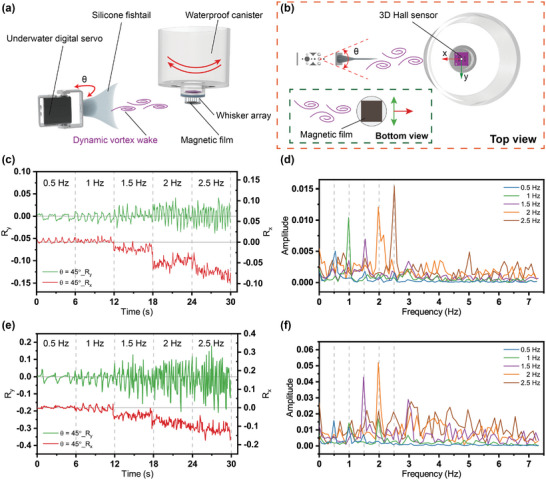
Frequency perception of dynamic vortex wake by the sensors. a) Experimental device of the dynamic vortex wake generator. b) Top view of the experimental device. c) The response of the whisker sensor to vortex wakes with different frequencies. d) Frequency‐domain signals of the whisker sensor in the *y* direction obtained by FFT (Fast Fourier Transform). e) The response of the cylinder array sensor to vortex wakes with different frequencies. f) Frequency‐domain signals of the cylinder array sensor in the y direction obtained by FFT. (The dashed lines represent the actual frequencies of the fishtail swing.).

The regular underwater movement of both organisms and non‐organisms (such as fishtail swing and blade rotation, etc.) will produce a dynamic vortex wake, which contains the motion and position information of the wake source. In addition to sensing the seal's own state of motion, their whiskers have a more important function, which is to track and hunt prey underwater. Therefore, seal whiskers are particularly sensitive to the perception of dynamic vortex wakes. Based on its special structure, our whisker sensor is also capable of sensing dynamic vortex wakes. As shown in **Figure** **4**a (Figure S2, Supporting Information), a silicone fishtail was driven by an underwater digital servo and swung regularly underwater, acting as a dynamic vortex wake generator. Our whisker sensor was still mounted on the bottom of the waterproof canister, which was fixed to the aluminum bracket and could still be rotated to change the relative direction of the wake source. In the demonstration experiments, the distance between the rotation shaft of the servo and the sensor was always 15 cm, and the servo drove the silicone fishtail at a certain frequency to swing regularly at angle θ (Figure 4b).

Figure 4c shows the responses of the whisker sensor to the vortex wakes generated by the swinging fishtail at different frequencies when θ   =  45°. The decoupling parameter signals *R_x_* and *R_y_* reflect the displacement changes of the magnetic film under the whisker array in the *x* direction and the y direction, respectively, thus indirectly reflecting the information of the vortex wakes. According to the signal *R_x_*, under the action of the vortex wakes generated by the swinging fishtail, the magnetic film shifted along the direction from the rotation shaft to the sensor. Meanwhile, the larger the swing frequency, the larger the displacement of the magnetic film shift. On the contrary, when the frequency was small (0.5 Hz), the magnetic film shifted toward the direction of the wake source, which was due to the negative pressure area caused by the discontinuous vortex wake generated by the pause of the servo when driving at low frequency. According to the signal *R_y_*, under the action of the vortex wakes generated by the swinging fishtail, the magnetic film periodically swung back and forth in a direction perpendicular to the direction from the rotating shaft to the sensor. We also conducted related experiments when θ   =  90°. (**Figure** S3a, Supporting Information) Because of the limited rotation speed of the digital servo, it could not maintain a large swing angle at a large swing frequency. Therefore, the output signals at 2.5 Hz were not recorded when θ  =  90°. From the comparison of the signals when θ  =  45° and θ  =  90°, the wake's strength immediately affected the swing amplitude, which also positively correlated with the swing angle of the fishtail but was not significantly impacted by the swing frequency. The discontinuous vortex wakes also contributed to the low‐frequency signal's relative weakening.

Signals *R_y_* at different frequencies and swing angles were processed by Fast Fourier Transform (FFT), respectively, obtaining the frequency‐domain signals of the whisker sensor, as shown in Figure 4d. When the swing angle θ is 45°, the peak amplitudes of the signal curves in the frequency domain obtained by FFT are 0.54, 0.99, 1.53, 1.97, and 2.51 Hz (main frequencies), respectively, which are the characteristic frequencies of the measured vortex wakes. The gray dashed lines in the figures represent the actual frequencies of the vortex wakes generated by the fishtail swing. The errors between them and the measured frequencies are within 0.04 Hz. The same is true for larger swing angles (Figure S3b, Supporting Information). However, the vortex wake signals perceived by the cylinder array sensor are rather messy, as shown in Figure 4e,f (Figure S3c, d, Supporting Information). The main frequencies obtained are 0.49, 1.97, 1.49, 1.97, and 3.08 Hz, respectively, with large errors in some frequencies. In addition, there are many non‐characteristic frequencies with similar amplitudes, which lead to inaccurate frequency identification. (See Figure S4, Supporting Information for Separate frequency‐domain signals of the sensors.) These results show that the whisker sensor is capable of perceiving the dynamic vortex wake frequency accurately. In addition, with the increase in the fishtail swing angle of the wake source strengthening the vortex wake, the peak amplitude at the main frequency of the frequency domain signal will increase, which makes the characteristic frequency of the signal easier to identify and calculate. Compared to the cylinder array sensor, the whisker sensor could reduce interference signals and obtain a clearer and more accurate wake frequency with its ability to suppress VIVs. The experimental findings show that for the artificial wake source, the vortex wake generated by the 45‐degree swing angle is powerful enough for our whisker sensor to detect it at a sensing distance of *D* = 15 cm. The sensor is adequate to perceive the characteristic frequencies of some of the weaker and harder‐to‐detect dynamic vortex wakes. However, the attenuation caused by the perceived distance may have more obvious effects on the intensity of the vortex wake and its perception, which is worthy of further research in our future work.

Characteristic frequency recognition allows the seal to sense the presence of prey, but it is the direction recognition of wakes that really endows the seal with the ability to track and capture prey. Our whisker sensor also has the ability to sense the direction of the dynamic vortex wake. According to the 2D displacement signals produced by the magnetic film under the action of the vortex wake in Figure 4c, the motion of the whisker array in the vortex wake can be separated into two parts: the symmetrical swing perpendicular to the direction from the wake source to the sensor and the swing in the direction from the wake source to the sensor with an offset. As a result, a new oscillating center is generated when the whisker array is exposed to the vortex wake, whose orientation relative to the origin is consistent with the direction of the sensor position relative to the wake source. Through this method, the direction perception of the dynamic vortex wake can be achieved by our sensor.

**Figure 5:**
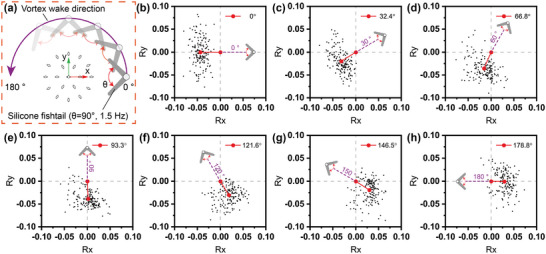
Direction perception of dynamic vortex wake by our sensor. a) Schematic diagram of the experimental method. Here, the angle and frequency of the silicone fishtail swing are 90° and 1.5 Hz, respectively. b–h) The 2D decoupling parameter signals of the sensor at different times when the dynamic vortex wake source is in the direction of 0°, 30°, 60°, 90°, 120°, 150°, and 180°, respectively.

**Figure** **5**a shows the experimental method for the multidirectional perception of vortex wakes. When the waterproof canister and the whisker sensor are rotated clockwise, the direction of the wake source relative to the sensor (vortex wake direction) will change from 0° to 180°, as shown in the schematic diagram. In this demonstration experiment, the silicone fishtail was swung at an angle of 90° and a frequency of 1.5 Hz to maintain a continuous vortex wake. The biaxial displacements of the magnetic film in the vortex wake were recorded at a frequency of ≈15 Hz, according to which the plane distribution of the oscillating position of the magnetic film could be obtained. Figure 5b–h shows the oscillating position distribution of the magnetic film in the vortex wake when the vortex wake direction is 0°, 30°, 60°, 90°, 120°, 150°, and 180°, respectively. The average of the coordinates of all the oscillating points in these distributions was calculated to get the coordinates of the oscillating center of the magnetic film, which were represented by red points and lines in the figures. The orientation of the origin point relative to the oscillation center is the direction of the wake source. The measured orientation of the wake source and the magnitude of its angle are indicated in the figures. Assuming that the measured direction of the 0‐degree wake is taken as the standard initial position (Figure 5b), the direction perception errors are 0°, 2.4°, 6.8°,3.3°, 1.6°, −3.5°, and −1.2°, respectively. Similarly, the perception errors of wake directions are also due to a number of factors, including the tilt of the seal whisker when glued, the angle deviation of the waterproof canister rotation, the influence of the pool wall, and so on. Consequently, the errors are acceptable, especially for the direction perception of complex multidirectional vortex wakes. As before, we conducted comparative experiments using the cylinder array sensor, as shown in Figure S5 (Supporting Information). Compared to the whisker sensor, the cylinder array sensor exhibits more pronounced oscillations in the same vortex wake. However, due to the inevitable manufacturing errors and the interference of VIVs, the measured directions of the vortex wake also show more significant deviations.

To sum up, compared to the cylinder array sensor, the whisker sensor has the stronger capability to accurately perceive the frequency of the dynamic vortex wake and effectively obtain the direction of the dynamic vortex wake, which in turn allows for the determination of the motion frequency and orientation of the vortex wake source relative to the sensor. Equipped with this capability, our sensor can be installed under an AUV to enable the AUV to perceive the motion frequency and orientation of both organisms and other non‐organisms, allowing it to identify and track them like seals.

## 3 Conclusion

The sensor proposed in this paper is a bio‐inspired hydrodynamic sensor with a centripetally magnetized film and whisker array architecture, which enables the detection of the underwater triaxial motion of the magnetic film, thus obtaining the relevant hydrodynamic information. The wireless penetration characteristic of magnetic sensing allows the sensor to be mounted separately on both sides of the bulkhead for underwater applications, ensuring the excellent watertightness of the equipment. The mechanism of the 3D self‐decoupling model was explained in detail, and the decoupling parameters were directly used to represent the triaxial motion of the magnetic film. Through the fluid‐structure coupling simulation, the arrangement and size of the whisker array were optimized, and the vortex‐induced vibration suppression ability of the whiskers at different angles of attack was verified. The fabrication and installation processes of the sensor were described in detail. The whisker sensor was compared with the cylindrical cilia array sensor in an annular flow field, demonstrating that our sensor was equipped with similar sensitivity and excellent vortex‐induced vibration suppression ability (RMSE < 0.061 m s^−1^), as well as good multidirectional sensing uniformity. Moreover, the whisker sensor was used to sense the vortex wake generated by a swinging silicone fishtail, which could not only accurately sense its frequency (error < 0.05 Hz) but also calculate and analyze the orientation of the wake source (error < 7°), thereby realizing the identification and tracking of underwater targets.

In summary, our sensor presents three unique innovations compared to existing miniaturized flow sensors.^[^ ^21^, ^22^, ^23^, ^34^, ^53^ ^]^ First, it features 3D self‐decoupling perception, enabling the sensor to acquire 2D hydrodynamic information similarly to a biological whisker, using a single sensing unit with an extremely simple structure and low cost, unlike other methods that require sensing unit arrays. Furthermore, the 3D self‐decoupling feature allows for adjustments to the initial installation position of the magnetic film without additional calibration, significantly facilitating the mass production and application of the sensor. Second, our sensor benefits from the vortex‐induced vibration suppression capability of the seal whisker array, which minimizes the impact of the whisker's own vibrations. This enhancement improves the stability of the sensor in receiving external flow field signals. Additionally, the centripetal arrangement of the whiskers reduces the anisotropic effects of the elliptic whisker structure, thereby enhancing the uniformity and stability of multidirectional perception. Third, the sensor's wireless penetration characteristic eliminates the need for leads and perforations, greatly improving its watertightness and pressure resistance in underwater applications, and making it suitable for deeper marine environments. These three innovations collectively enhance the performance of our sensor and expand its potential applications, providing a novel solution for the perception and cluster communication of underwater robots (Table S3, Supporting Information).

However, considering future integrations and applications, there are still many improvements that can be made to the sensor. First of all, in order to facilitate fabrication and demonstration, the sensor in this paper has a large size, which can be reduced to meet the requirements in practical applications. Second, the magnetic film is located at the end of the sensor, whose own gravity will not only reduce the sensing sensitivity of the sensor but also be prone to limiting the installation angle and position of the sensor. In future work, the influence of gravity may be balanced by changing the position of the magnetic film. In conclusion, this paper reports a biomimetic whisker sensor based on magnetic 3D self‐decoupling for the perception of the multidirectional steady‐state flow field and dynamic vortex wake, which is equipped with a multidimensional perception capability similar to actual seal whiskers. In the future, our sensor will be applied to underwater robots and submersibles through miniaturization and arraying to complete important underwater tasks such as motion perception, assisted navigation, environmental information measurement, and cluster communication.

## 4 Experimental Section

### Preparation of Magnetic Materials

The raw material of the magnetic film was composed of the following ingredients: 11.71 wt.% SE 1700 base (DOWSIL), 1.17 wt.% SE 1700 catalyst (DOWSIL), 21.78 wt.% Ecoflex 00–30 Part B (Shanghai Zhixin Corp., Shanghai, China), 2.72 wt.% fumed silica nanoparticles (Aladdin Biochemistry Technology Corp., Shanghai, China), and 62.62 wt.% NdFeB microparticles (Jianghuai Ciye Corp., Huai'an, China).^[^ ^42^ ^]^ SE1700 was the hard elastomer substrate, while Ecoflex 00–30 Part B was a softer one used to achieve the better mechanical properties of composite material. Fumed silica nanoparticles were added to achieve the required rheological properties for molding.

### Fabrication of Magnetic Films

The magnetic microparticles were added to the elastomer mixture and mixed by a planetary mixer at 2000 rpm. for 2 min, followed by defoaming at 2000 rpm for 2 min. As shown in Figure S6a (Supporting Information), the fully mixed magnetic paste was added between two pieces of tempered glass, which were coated with the release liner, and then molded into a film by extrusion with two gaskets to control the thickness (1 mm). The film was then heated at 120 °C for 1.5 h to fully cure. After curing, the film was cut into squares, which were folded into arrowheads and inverted in a 3D‐printed fixture. The square films were then magnetized by impulse magnetic fields (≈3.6 T) generated by an impulse magnetizer (JH12160, Jiuheng Huisheng Magnetoelectric Technology Corp., Ningbo, China) to obtain the centripetal magnetization arrangement.

### Fabrication of the Whisker Array Embedded with a Magnetic Film

The magnetized magnetic film was redeployed and embedded in the 3D‐printed mold base, as shown in Figure S6b (Supporting Information). The magnetic film was raised by the small cylinders (height: 0.2 mm; radius: 0.6 mm) in the base, ensuring that the magnetic film could be completely wrapped after the silicone injection to avoid the bending of the magnetic film caused by the unilateral silicone solidification and contraction. After fixing the mold of “seal whiskers” to the mold base, Ecoflex 00–30 was poured through the holes above the mold (Figure S6c, Supporting Information), which was then defoamed by vacuum to ensure that the silicone elastomer filled all gaps in the mold. After curing at 50 °C for 2 h, the mold was reopened, and the “seal whiskers” array elastomer embedded with the magnetic film was demolded (Figure S6d, Supporting Information). The Young's modulus of the elastomer material was 0.074 MPa, while the Poisson's ratio was 0.48. (Figure S7, Supporting Information)

### Assembly of the Biomimetic Hydrodynamic Sensor

As shown in Figure S6e (Supporting Information), a 3D‐printed detachable base was then evenly coated with a layer of silicone glue (DL‐1020, Dinglifeng Technology Corp., Shenzhen, China, fully cured after 24 h at room temperature. Thickness: 0.5 mm;), which was used to attach the elastomer to the detachable base. The detachable base included upper and lower parts (blue part and grey part), both of which could be quickly installed and detached by mortise and tenon structure (thickness 4 mm, Figure S6f, Supporting Information) to facilitate the replacement of sensor samples in experiments. The upper part of the base and the Hall sensor were glued on two sides of the bulkhead, respectively, as shown in Figure S6g (Supporting Information). In this process, the alignment of the Hall sensor's center and the magnetic film's center could be achieved by measuring the position with the maximum flux density in the *z*‐direction (perpendicular to the magnetic film plane), which was not necessary because, benefiting from the 3D self‐decoupling model, the initial position of the magnetic film could be calculated and its influence could be excluded. During the experiment, the detachable base and the seal whisker array elastomer were underwater and subjected to hydrodynamic action, while the Hall sensor was in the dry cabin and wirelessly sensed the triaxial displacements of the magnetic film. Consequently, there were no wires or holes in the bulkhead, completely avoiding possible watertight problems.

## Conflict of Interest

The authors declare no conflict of interest.

## Author Contributions

H.D. led the development of the concepts, designed the experiments, analyzed the data, and interpreted the results. C.Z. conceived the idea, designed the experiments, and revised the paper and figures. H.H., Z.H., H.S., D.T., and C.L. partially participated in the experiments and measurements. T.L., J.F., P.Z., and H.Y. conceived the idea. C.Z. and P.Z. provided funding support. H.D. and C.Z. performed the theoretical studies, prepared the figures, and wrote the manuscript. All authors participated in the discussion of this project.

## Supporting information

Supporting Information

## Data Availability

The data that support the findings of this study are available from the corresponding author upon reasonable request.
