# Supplementary material for: Biomimetic Hydrodynamic Sensor with Whisker Array Architecture and Multidirectional Perception Ability
Source: Adv Sci (Weinh). 2024 Aug 9;11(38):2405276. doi: 10.1002/advs.202405276 (PMC11481291; doi:10.1002/advs.202405276)
Supplement: Supplementary file 1 — Supporting Information [file ADVS-11-2405276-s001.docx]

**Supporting Information**

**Biomimetic** **hydrodynamic sensor with whisker array architecture and multidirectional perception ability**

*Huangzhe Dai^1,2^, Chengqian Zhang^1*^, Hao Hu^1,2^, Zhezai Hu^1,2^, Haonan Sun^1,2^, Kan Liu^1^,* *Tiefeng Li^1,3^, Jianzhong Fu^1,2^, Peng Zhao^1,2^, and Huayong Yang^1^*

*(^1^* *The State Key Laboratory of Fluid Power and Mechatronic Systems, College of Mechanical Engineering, Zhejiang University, Hangzhou 310027, China*

*^2^ The Key Laboratory of 3D Printing Process and Equipment of Zhejiang Province, College of Mechanical Engineering, Zhejiang University, Hangzhou 310027, China*

*^3^ Center for X-Mechanics, Department of Engineering Mechanics, Zhejiang University, 310027, Hangzhou, China*

*^*^ Corresponding Author Email: zhangcq@zju.edu.cn)*

**The PDF file includes:**

Supplementary Table S1. Parameters of the idealized whisker surface model.

Supplementary Table S2. Parameters of the finalized whisker array sensor.

Supplementary Table S3. Comparison of state-of-the-art hydrodynamic sensors, measurement dimensions and decoupling methods.

Supplementary Figure S1. Experimental photo of the annular steady-state flow field generator and the water tank.

Supplementary Figure S2. Experimental photo of the dynamic vortex wake generator.

Supplementary Figure S3. Frequency perception of dynamic vortex wake by the sensors when the angle of the silicone fishtail swing is 90°.

Supplementary Figure S4. Separate frequency-domain signals of the sensors in the y direction obtained by FFT.

Supplementary Figure S5. Direction perception of dynamic vortex wake by the cylinder array sensor.

Supplementary Figure S6. Fabrication and installation of the whisker sensor.

Supplementary Figure S7. The elastic properties of the whisker array.

Supplementary Reference

**Supplementary Table S1.** Parameters of the idealized whisker surface model.

| Parameter | Meaning | Idealized value^[1]^ | Value in this work |
| --- | --- | --- | --- |
| M | The half period of the undulation | 0.91 mm | 1.82 mm |
| a | The semi-major axis of the cross-section in the laterally wide location | 0.595 mm | 1.19 mm |
| b | The semi-minor axis of the cross-section in the laterally wide location | 0.24 mm | 0.48 mm |
| c | The semi-major axis of the cross-section in the laterally narrow location | 0.475 mm | 0.95 mm |
| d | The semi-minor axis of the cross-section in the laterally narrow location | 0.29 mm | 0.58 mm |
| $\alpha$ | The angle of the connecting line between the maxima of the undulation on the rostral and caudal edges of the whisker | 15.27 deg | 15.27 deg |
| $\beta$ | The angle of the connecting line between the minima of the undulation on the rostral and caudal edges of the whisker | 17.60 deg | 17.60 deg |

**Supplementary Table S2.** Parameters of the finalized whisker array sensor.

| Component | Parameter | Value  [mm] |
| --- | --- | --- |
| Magnetic film | Side length | 20 |
|  | Thickness | 1 |
| Whisker array | Thickness of bottom layer ^a)^ | 1.4 |
|  | Length of the whisker | 10.92 |
| Detachable base | Thickness | 5 |
| Bulkhead | Thickness | 3 |
| MLX90393 | Size | $3\times3\times1$ |
| / | Distance between the magnetic film and the Hall sensor | 19.12 |

1. The magnetic film is embedded in the middle of the bottom silicone layer of the whisker array.

**Supplementary Table S3.** Comparison of state-of-the-art hydrodynamic sensors, measurement dimensions and decoupling methods.

| Sensor type | | Sensor output | | | | | Other feature | |
| --- | --- | --- | --- | --- | --- | --- | --- | --- |
|  |  | Decoupling method | Flow velocity | Flow direction | Wake frequency | Wake direction | Transmission mode | Natural watertightness |
| **Piezoresistive** | Artificial lateral line^[2]^ | Sensor array | Yes | Yes | No | No | Wired transmission | No |
|  | 3D printed graphene sensor^[3]^ | NA | No | No | Yes | No | Wired transmission | No |
|  | Ultraflexible flow sensor^[4]^ | NA | Yes | No | Yes | No | Wired transmission | No |
|  | Flexible airflow Sensor^[5]^ | Sensor array | Yes | Yes | No | No | Wireless transmission | No |
|  | Artificial whisker sensor^[6]^ | Sensor array | Yes | Yes | Yes | No | Wired transmission | No |
| **piezoelectric** | Whisker inspired piezoelectric sensor^[7]^ | NA | Yes | Yes | Yes | No | Wired transmission | No |
| **Pressure** | Distributed sensing for fluid disturbance compensation^[8]^ | Sensor array | Yes | Yes | No | No | Wired transmission | No |
|  | Artificial  lateral line (pressure sensor)^[9]^ | Sensor array | Yes | Yes | No | No | Wired transmission | No |
| **Triboelectric** | Underwater bionic whisker sensor^[10]^ | NA | No | No | Yes | No | Wired transmission | No |
| **Magnetic** | Actuation-enhanced artificial cilia^[11]^ | NA | Yes | No | No | No | Wired transmission | No |
|  | Biomimetic flow  sensing^[12]^ | NA | Yes | No | No | No | Wireless transmission | Yes |
|  | **This work** | **Formula derivation** | **Yes** | **Yes** | **Yes** | **Yes** | **Wireless transmission** | **Yes** |


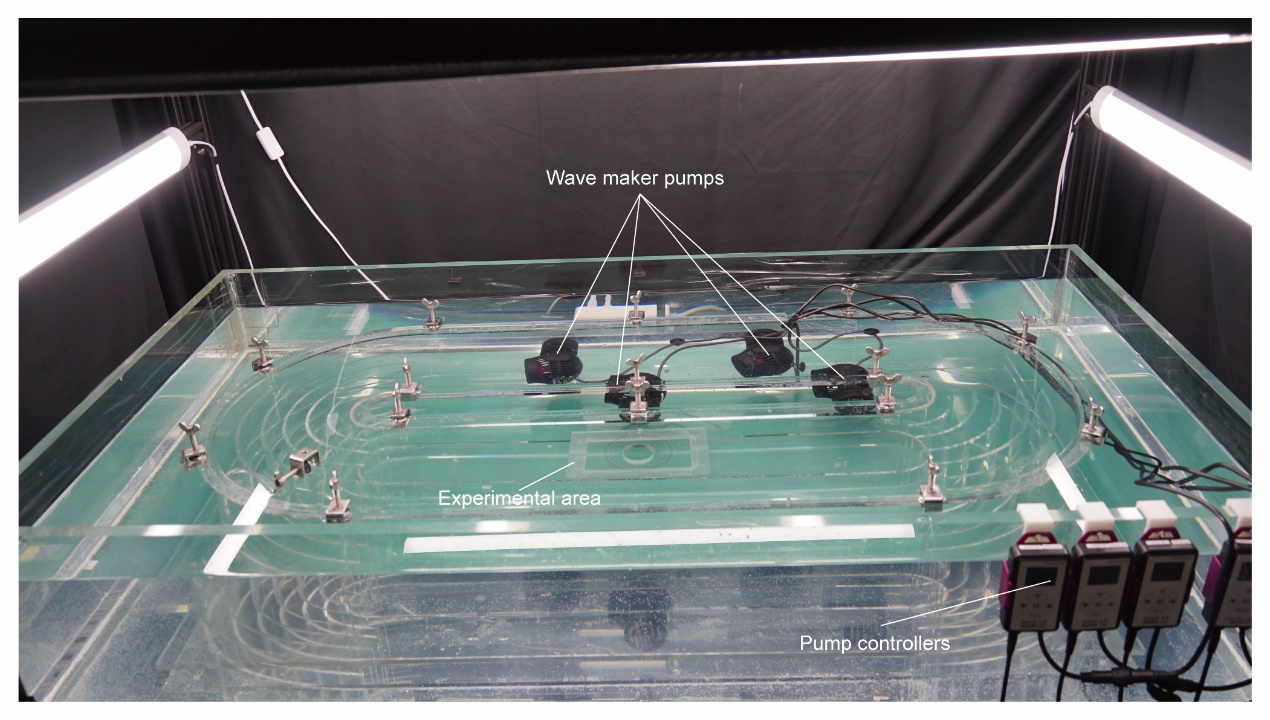


**Supplementary Figure S1.** Experimental photo of the annular steady-state flow field generator and the water tank


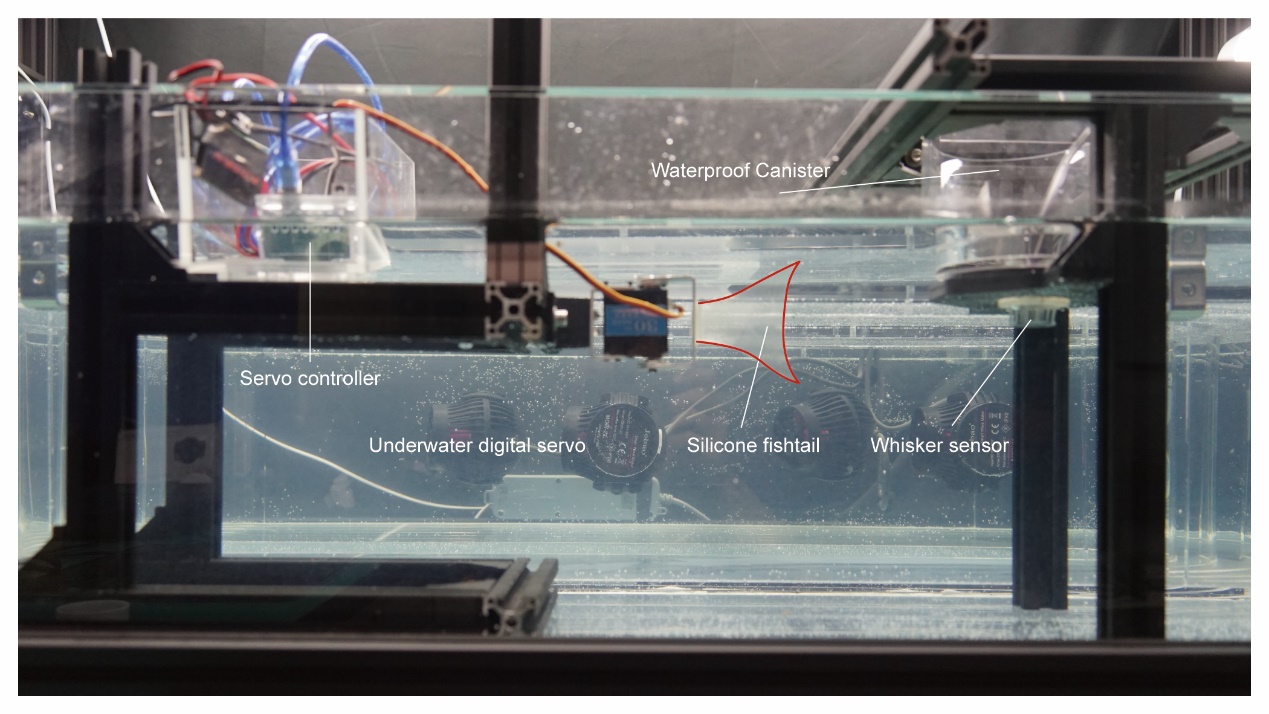


**Supplementary Figure S2.** Experimental photo of the dynamic vortex wake generator.


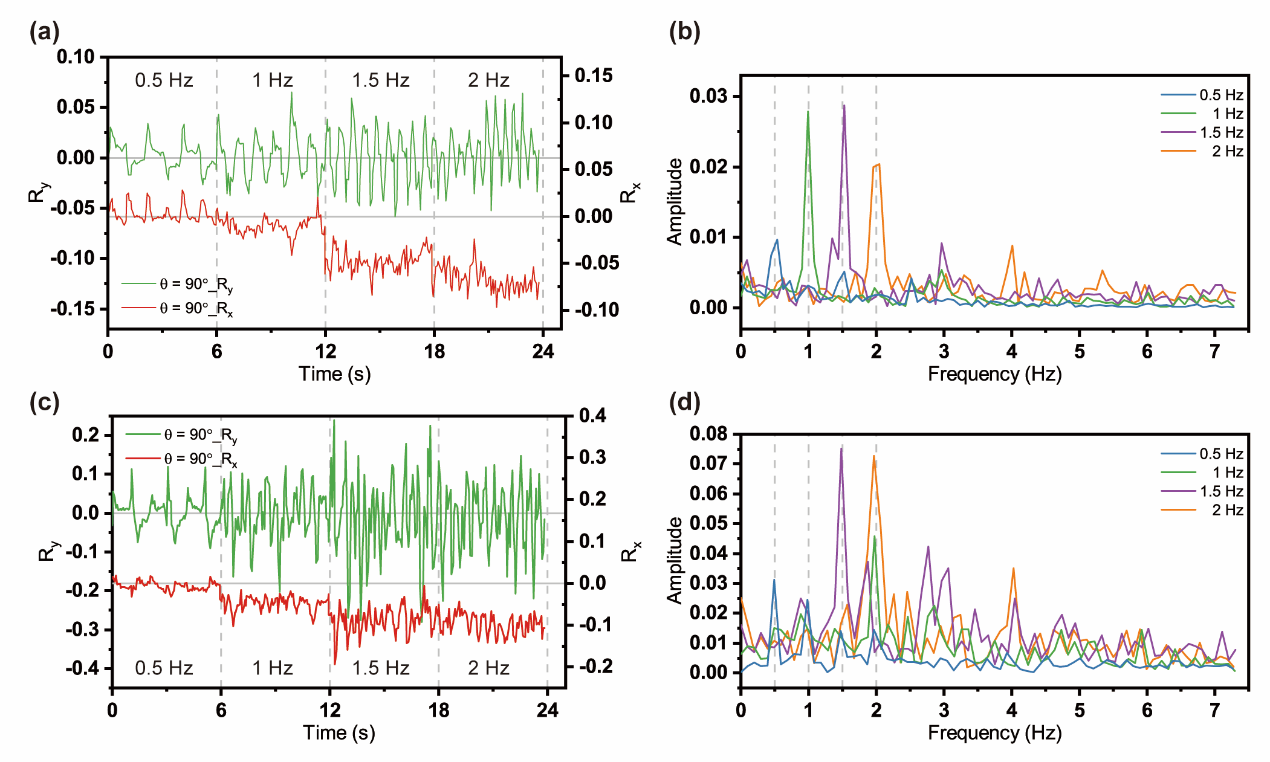


**Supplementary Figure S3.** Frequency perception of dynamic vortex wake by the sensors when the angle of the silicone fishtail swing is 90$^{\circ}$. a) The response of the whisker sensor to vortex wakes with different frequencies. b) Frequency-domain signals of the whisker sensor in the y direction obtained by FFT (Fast Fourier Transform). c) The response of the cylinder array sensor to vortex wakes with different frequencies. d) Frequency-domain signals of the cylinder array sensor in the y direction obtained by FFT. (The dashed lines represent the actual frequencies of the fishtail swing.)


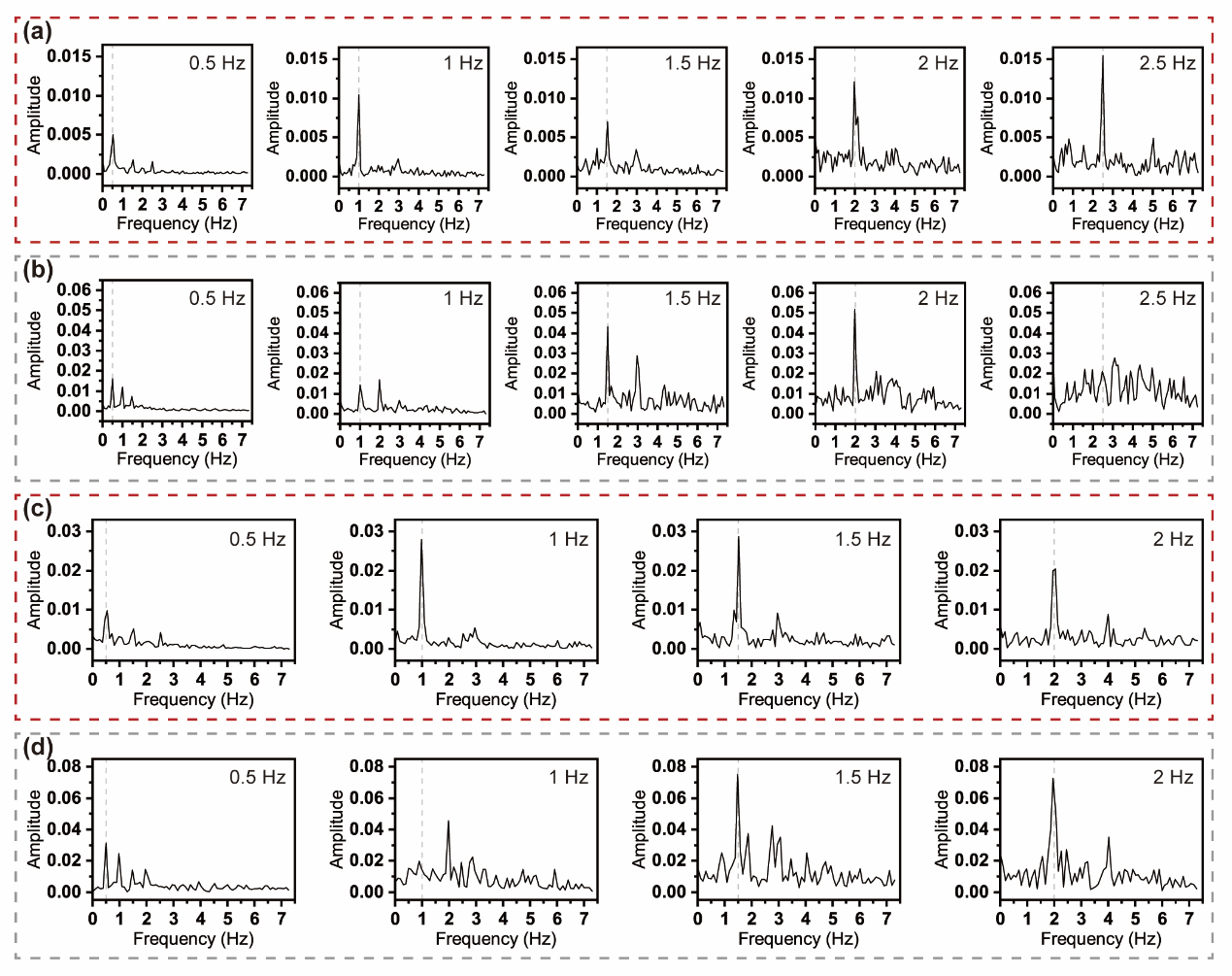


**Supplementary Figure S4.** Separate frequency-domain signals of the sensors in the y direction obtained by FFT. a) Separate frequency-domain signals of the whisker sensor when the angle of the silicone fishtail swing is 45$^{\circ}$. b) Separate frequency-domain signals of the cylinder array sensor when the angle of the silicone fishtail swing is 45$^{\circ}$. c) Separate frequency-domain signals of the whisker sensor when the angle of the silicone fishtail swing is 90$^{\circ}$. d) Separate frequency-domain signals of the cylinder array sensor when the angle of the silicone fishtail swing is 90$^{\circ}$.


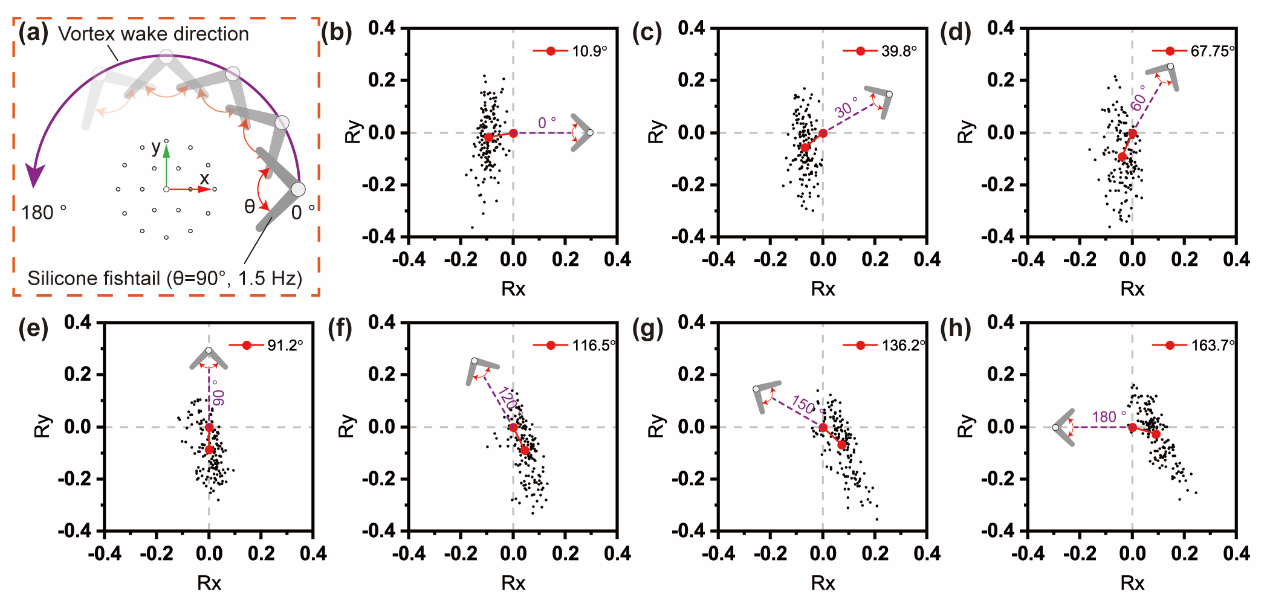


**Supplementary Figure S5.** Direction perception of dynamic vortex wake by the cylinder array sensor. a) Schematic diagram of the experimental method. Here, the angle and frequency of the silicone fishtail swing are 90° and 1.5Hz, respectively. b-h) The 2D decoupling parameter signals of the sensor at different times when the dynamic vortex wake source is in the direction of 0°, 30°, 60°, 90°, 120°, 150°, and 180°, respectively.


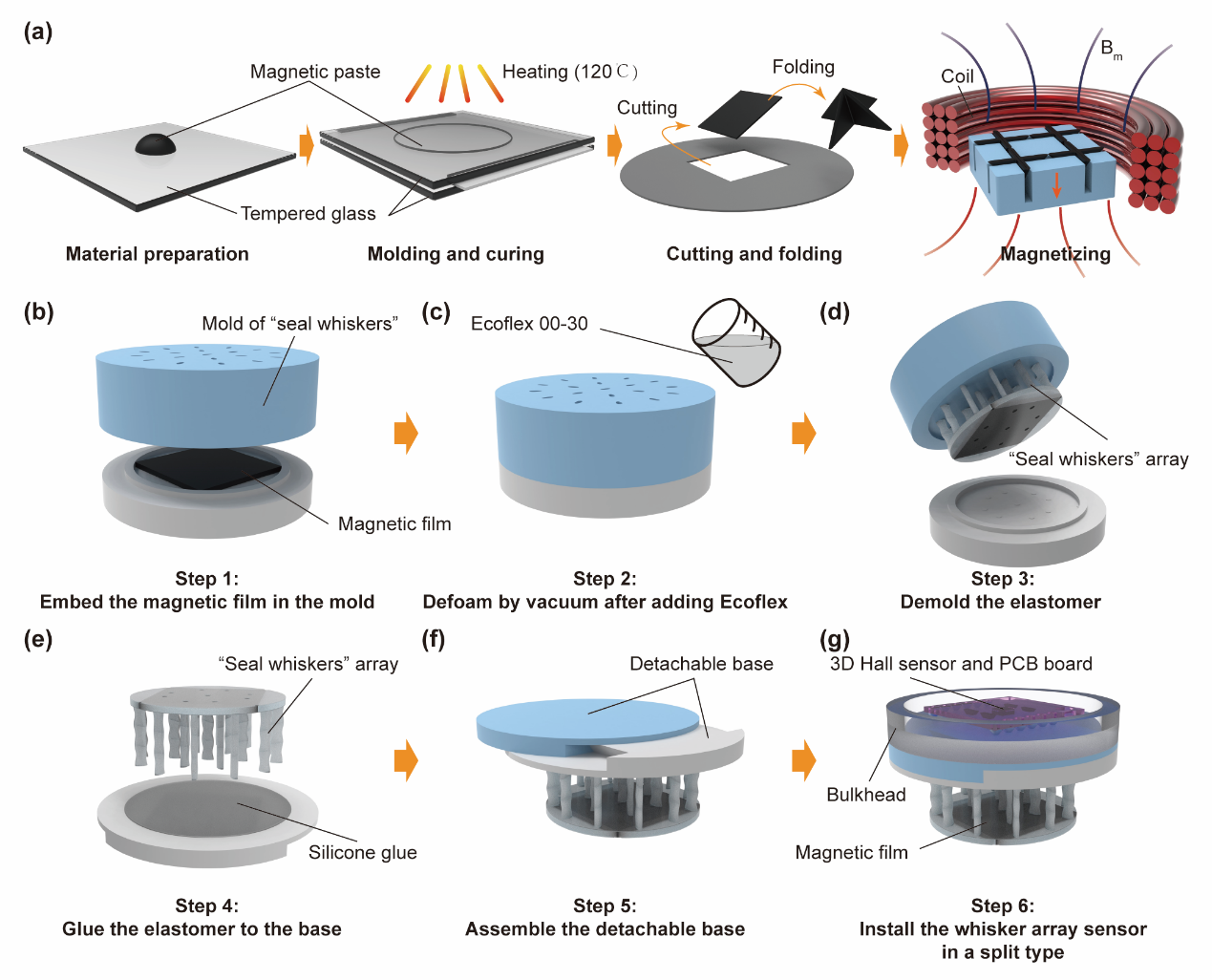


**Supplementary Figure S6.** Fabrication and installation of the whisker sensor. a) Preparation of the centripetally magnetized magnetic film. b) Step 1: Embed the magnetic film in the mold base of the "seal whiskers" array. c) Step 2: Defoam by vacuum after adding Ecoflex 00-30 and wait for it to cure completely. d) Step 3: Demold the "seal whiskers" array elastomer. e) Step 4: Glue the elastomer to the detachable base with the silicone glue. f) Step 5: Assemble the detachable base. g) Step 6: Glue the Hall sensor and the detachable base (step 5) on two sides of the bulkhead, respectively.


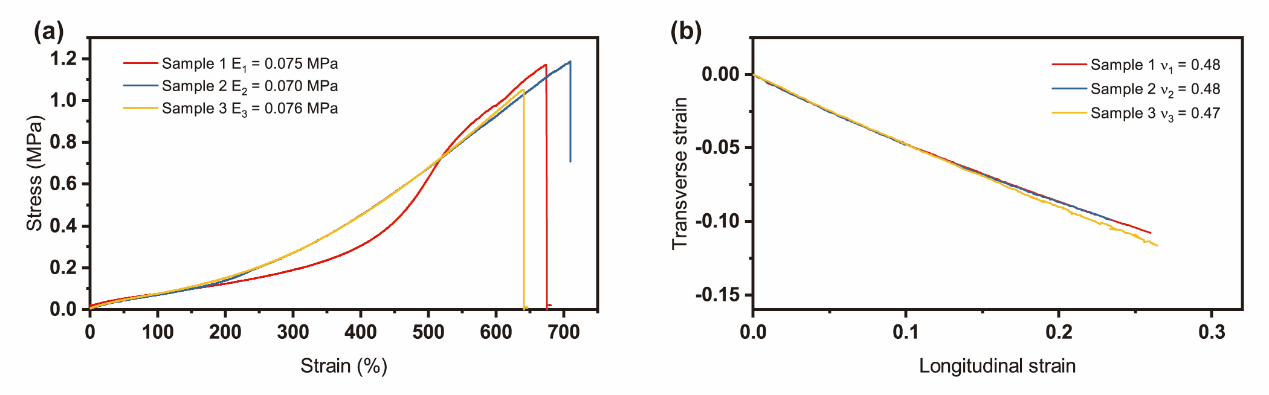


**Supplementary Figure S7.** The elastic properties of the whisker array. a) Relationships between stress and strain of the elastomer materials. b) Relationships between transverse strain and longitudinal strain of the elastomer materials.

**Reference**

[1] W. Hanke, M. Witte, L. Miersch, M. Brede, J. Oeffner, M. Michael, F. Hanke, A. Leder, G. Dehnhardt, *J Exp Biol* **2010**, *213*, 2665.

[2] Z. Zhang, C. Zhou, L. Cheng, X. Wang, M. Tan, *IEEE transactions on robotics* **2023**, *39*, 1.

[3] X. Zheng, A. M. Kamat, A. O. Krushynska, M. Cao, A. G. P. Kottapalli, *Advanced Functional Materials* **2022**, *32*, 2207274.

[4] S. Abolpour Moshizi, H. Moradi, S. Wu, Z. J. Han, A. Razmjou, M. Asadnia, *Advanced Materials Technologies* **2022**, *7*, 2100783.

[5] D. Shen, Y. Jiang, Z. Ma, P. Zhao, Z. Gong, Z. Dong, D. Zhang, *Journal of bionics engineering* **2022**, *19*, 73.

[6] G. Liu, Y. Jiang, P. Wu, Z. Ma, H. Chen, D. Zhang, *Soft Robot* **2023**, *10*, 97.

[7] X. Zhang, X. Shan, T. Xie, J. Miao, H. Du, R. Song, *Measurement* **2021**, *172*, 108866.

[8] M. Krieg, K. Nelson, K. Mohseni, *Nature machine intelligence* **2019**, *1*, 216.

[9] X. Zheng, W. Wang, M. Xiong, G. Xie, *IEEE Transactions on Robotics* **2020**, *36*, 472.

[10] S. Wang, P. Xu, X. Wang, J. Zheng, X. Liu, J. Liu, T. Chen, H. Wang, G. Xie, J. Tao, M. Xu, *Nano Energy* **2022**, *97*, 107210.

[11] J. Han, X. Dong, Z. Yin, S. Zhang, M. Li, Z. Zheng, M. C. Ugurlu, W. Jiang, H. Liu, M. Sitti, *Proc Natl Acad Sci U S A* **2023**, *120*, e1986666176.

[12] A. Alfadhel, B. Li, A. Zaher, O. Yassine, J. Kosel, *Lab Chip* **2014**, *14*, 4362.
